# Spatial visualization of drug uptake and distribution in *Fasciola hepatica* using high-resolution AP-SMALDI mass spectrometry imaging

**DOI:** 10.1007/s00436-021-07388-1

**Published:** 2022-01-24

**Authors:** Carolin M. Morawietz, Alejandra M. Peter Ventura, Christoph G. Grevelding, Simone Haeberlein, Bernhard Spengler

**Affiliations:** 1 Institute of Inorganic and Analytical Chemistry, Justus Liebig University Giessen, Giessen, Germany; 2 Institute of Parasitology, Biomedical Research Center Seltersberg (BFS), Justus Liebig University Giessen, Giessen, Germany

**Keywords:** Mass spectrometry imaging, *Fasciola hepatica*, Triclabendazole, Triclabendazole sulfoxide, Drugs

## Abstract

Understanding drug penetration, distribution, and metabolization is fundamental for understanding drug efficacy. This also accounts for parasites during antiparasitic treatment. Recently, we established matrix-assisted laser desorption/ionization (MALDI) mass spectrometry imaging (MSI) in blood flukes and liver flukes. This label-free technique is capable of visualizing the molecular distribution of endogenous and exogenous molecules, such as drug compounds. Here, we conducted atmospheric-pressure scanning microprobe MALDI MSI (AP-SMALDI MSI) of tissue sections of adult *Fasciola hepatica* that have been treated in vitro with 100 µM of triclabendazole (TCBZ), the drug of choice for treatment of fasciolosis, and its main metabolite triclabendazole sulfoxide (TCBZ-SO). Measurements covered an *m*/*z* mass range of 250–1,000 and provided a high spatial resolution using a pixel size of 10 µm. To support the interpretation of drug distribution, we first identified endogenous lipids that mark characteristic tissues such as the gastrodermis, the tegument, and the parenchyma. The obtained results suggested an early tegumental route of TCBZ uptake within 20 min, followed by spreading throughout the parasite after 4 h, and an even distribution in most tissues after 12 h. This coincided with a strong reduction of parasite vitality. TCBZ-SO treatment demonstrated the accumulation of this metabolite in the same tissues as the parent drug compound. These data demonstrate the auspicious potential of MALDI MSI to visualize uptake and distribution patterns of drugs or drug-candidate compounds in parasites, which might contribute to preclinical drug discovery in liver fluke research and beyond.

**Supplementary Information:**

The online version contains supplementary material available at 10.1007/s00436-021-07388-1.

## Introduction

Triclabendazole remains the drug of choice for the control of fasciolosis in humans and livestock, particularly because of its high efficacy against both juvenile and adult stages. However, its intense and widespread use has led to the spread of resistance in numerous countries (Fairweather et al. 2020; Webb und Cabada 2018). Finding alternative treatment options is highly demanded, and several investigators are engaged in developing and testing novel compounds as drug candidates against liver flukes such as *Fasciola hepatica* (Edwards et al. 2015; Machicado et al. 2019; Morawietz et al. 2020; O'Neill et al. 2015; Kirchhofer et al. 2012). Understanding how drugs distribute and accumulate in this parasite is important to reveal (i) whether the uptake route is oral or via its surface, (ii) to answer how fast uptake occurs into the parasite, (iii) how this relates to the onset of vitality loss, (iv) to add knowledge on the mode of action and if the drug accumulates in particular parasite organs, and (v) to study the metabolization of a drug within the parasite, e.g., to bioactive or inactive products. Yet, while drug imaging is an established methodological approach in many fields of modern human pharmaceutical R&D and preclinical drug discovery, it is still in its infancy in the field of parasitology.

Conventional methods that localize drugs in tissue samples include immunofluorescence microscopy and positron emission tomography (PET). These techniques have limitations as they require the addition of fluorescent or radioactive labels to the drug that can potentially affect its molecular and in situ behavior (Piel et al. 2014; Ding et al. 2013). In addition, the spatial resolution of PET is limited to the low millimeter-range making drug distribution studies on such small samples impossible (Watakabe et al. 2020). Matrix-assisted laser desorption/ionization (MALDI) mass spectrometry imaging (MSI) is a cutting-edge method in the fields of life sciences and biomedicine that we recently adopted and optimized for the purpose of drug imaging in trematode parasites (Mokosch et al. 2021; Morawietz et al. 2020). MALDI MSI is a powerful technique that integrates molecular imaging and advanced image analysis. Thereby, it can visualize the molecular distribution of a large number of endogenous and exogenous compounds simultaneously in tissues without the addition of labels (Schulz et al. 2019; Römpp und Spengler 2013). When applied as part of preclinical drug discovery, this method provides important information about how a therapeutic substance penetrates, distributes, and metabolizes in (parasite) tissues. The technique involves the spot-wise desorption and ionization of the surface molecules of a tissue section using a pulsed UV laser beam. A mass spectrum is acquired for every ionized sample spot or “pixel “. Finally, by computational analysis, a pixel map is reconstructed that allows the spatial visualization of virtually any ionized molecule within the tissue section (Spengler 2015).

Here, we conducted AP-SMALDI MSI analyses to determine the spatial distribution of TCBZ and one of its main metabolites, TCBZ sulfoxide (TCBZ-SO), within *F. hepatica* after in vitro exposure to the drug compounds for different time periods.

Our approach delivered answers about the route and kinetic of drug uptake as well as its tissue tropism. To support the interpretation of drug distribution in MSI images, we additionally identified endogenous lipids that mark individual tissues such as the gastrodermis. Methodological points to consider and address in our study are the type of molecule desired to detect (is it ionizable?), whether to conduct the MSI measurement in negative- or positive-ion mode, to confirm the detectability and detection limit of the molecule by analysis of standard solutions, to choose the most suitable type of matrix for in-tissue detection, and to confirm the specificity of the detected *m*/*z* signal for the molecule of interest, e.g., by root-mean-square-error (RMSE) plot analysis.

## Materials and methods

### Experimental animals and parasites

Male Wistar rats RjHan:WI (*Rattus norvegicus*; Janvier, France) were used as model hosts to obtain adult stages of *F. hepatica*. Rats at 5 weeks age were orally infected with 20–25 metacercariae of an Italian parasite strain (Ridgeway Research, UK), which is TCBZ-susceptible. Adult flukes were collected from bile ducts at 10–12 weeks p.i. Worms were kept for at least 1 h in Roswell Park Memorial Institute Medium (RPMI 1640, Gibco, Thermo Fisher Scientific, Bremen, Germany) prior to use in experiments to allow clearance of gut contents.

### Drug treatment, embedding, and sectioning of *F. hepatica for AP-SMALDI MSI*

Adult *F. hepatica* were treated with triclabendazole (TCBZ) for three different time periods (20 min, 4 h, and 12 h) or with TCBZ-sulfoxide (TCBZ-SO) for 12 h (both from Sigma-Aldrich, Supelco, VETRANAL®, analytical standard, USA). We favored in vitro over in vivo treatment in order to precisely adjust drug concentration and exposure time of the parasite. The treatment was performed with two biological replicates for every exposure time period. Both drugs were used at 100 µM in RPMI medium supplemented with 5% chicken serum and 1% ABAM-solution [10,000 units penicillin, 10 mg streptomycin and 25 mg amphotericin B per ml] (all from Sigma-Aldrich, Gibco). Subsequent to the drug treatment, worms were successively washed in PBS solution and distilled water for about 1 min each. Then, they were placed in a Tissue-Tek Cryomold (15 × 15 × 5 mm^3^, Sakura Finetek, Netherlands) containing liquid aqueous gelatine solution of 8 wt% (gelatine powder, VWR, USA) and frozen on dry ice. Until further use, the worm samples were stored at − 80 °C. Transversal sections (20-µm thickness) of the embedded *F. hepatica* were prepared in a cryostat HM525 (Thermo Fisher Scientific) at around − 23 °C. Section quality was monitored using a digital light microscope (VHX-5000, Keyence, Japan). After quality control, the sections were stored at − 80 °C until AP-SMALDI MSI analysis.

### AP-SMALDI MSI sample preparation and measurements

Prior to matrix application, the worm sections were thawed and protected from humidity in a desiccator for 30 min. For the TCBZ-treated sections, a matrix solution consisting of 2,5 dihydroxybenzoic acid (DHB, for synthesis, Merck, Germany) was used in a concentration of 30 g/L in acetone/water 1:1 *v*:*v* (acetone uvasol, Merck; water HiPerSolv Chromanorm for HPLC, filtered at 0.2 µm, VWR) with addition of 0.1 vol% of trifluoroacetic acid (TFA, uvasol for spectroscopy, Merck). For the TCBZ-SO-treated section, α-cyano-4-hydroxycinnamic acid (CHCA, purity 97%, Sigma-Aldrich, USA) was used as a matrix due to better signal compared to DHB. The matrix solution was prepared in a concentration of 7 g/L in acetone/water 1:1 *v*:*v* with addition of 0.1 vol% TFA. Volumes of 100 µL in the case of DHB and 90 µL in the case of CHCA were sprayed onto the section surface using an ultrafine pneumatic sprayer system (SMALDIPrep, TransMIT GmbH, Giessen, Germany) with a flow rate of 10 µL/min and an N_2_-pressure of 1 bar. All matrix solutions were prepared freshly before each AP-SMALDI MSI measurement. AP-SMALDI MSI was performed on a Q Exactive HF orbital trapping mass spectrometer (Scheltema et al. 2014) (Thermo Fisher Scientific) equipped with an autofocusing AP-SMALDI5 AF ion source (Kompauer et al. 2017a, b) (TransMIT GmbH). Using the pixel-wise autofocusing feature of the instrument, the measurements were executed with a step size of 10 µm and 50 laser pulses per pixel. All imaging experiments were performed in positive-ion mode with a mass resolution of 240,000 at *m*/*z* 200. An *m*/*z* mass range of 250–1,000 was chosen for all measurements. For internal calibration with DHB, the lock masses *m*/*z* 273.03937 (corresponding to [2DHB + H − 2H_2_O]^+^) and *m*/*z* 716.12462 (corresponding to [5DHB − 4H_2_O + NH_4_]^+^) were set. For the measurement with CHCA, a lock mass of *m*/*z* 401.07441 (corresponding to [2CHCA + Na]^+^) was used. Furthermore, a maximum ion injection time of 500 ms, an S-lens RF level of 100 arbitrary units, a capillary temperature of 250 °C, and an acceleration voltage of 3.00 kV were adjusted.

### Data acquisition and analysis of AP-SMALDI MSI experiments

For controlling the AP-SMALDI ion source, SMALDIControl software (V1.1–118, TransMIT GmbH) was applied. Data acquisition was executed using Q Exactive Tune (version 2.9, Thermo Fisher Scientific). XCalibur (version 4.0.27.13, Thermo Fisher Scientific) was utilized for processing of the mass spectra. Visualization of imaging data was achieved using Mirion software package (Paschke et al. 2013) (version 3.3.64.20, TransMIT GmbH), and the bin width was adjusted to 0.005 u. No TIC normalization was applied in the image creation process. Assignment of the compounds was based on the accurate mass values with a maximum deviation of 3 ppm and LIPIDMAPS (Fahy et al. 2007) database results. For further consolidation, root-mean-square-error (RMSE) plots were created within the Mirion software package.

### H&E-staining after AP-SMALDI MSI

Subsequently to the imaging experiments, the sample surface was rinsed with 80 vol% aqueous ethanol (EtOH purissimum, Roth) to remove the matrix layer. Staining was conducted according to the hematoxylin and eosin (H&E) staining protocol (Mayer’s Hematoxylin and Eosin-Y solution, Sigma-Aldrich; Eukitt quick hardening mounting medium for microscopy, Honeywell-Fluka). After drying of the mounting medium, optical images of 250 × magnification were acquired by use of a digital light microscope (VHX-5000, Keyence).

## Results and discussion

### Optimal sectioning of *F. hepatica* for MALDI MSI

**Fig. 1:**
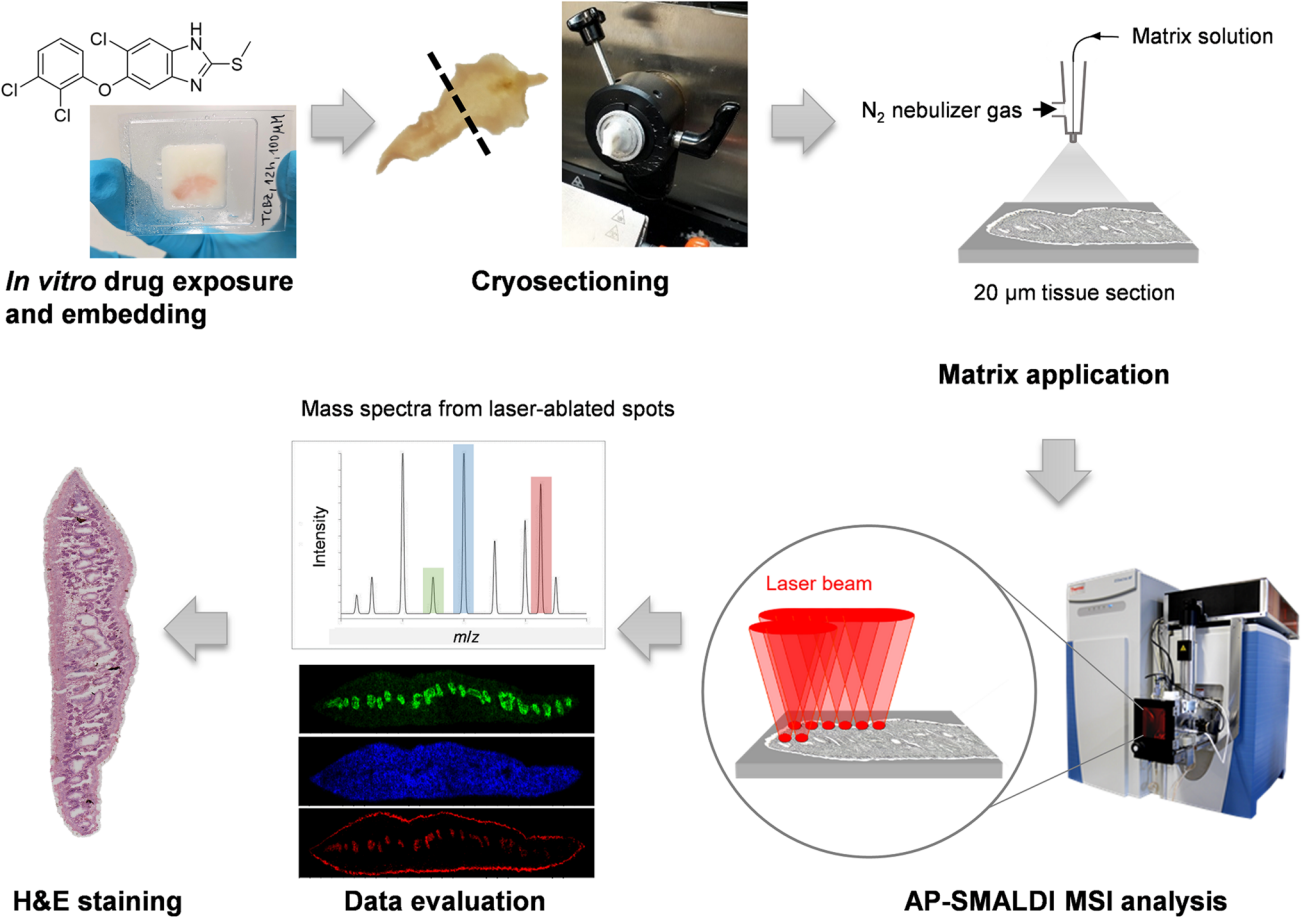
General workflow for drug imaging and lipid analysis with AP-SMALDI MSI in *F. hepatica*. After drug exposure and embedding of the liver fluke in gelatine solution, the sample is sectioned transversally in a cryotome. Matrix solution is deposited on the 20-µm-thick section with the help of an N_2_ gas stream in an automated system, followed by the AP-SMALDI MSI measurement where a laser beam ablates sample material in a rasterized manner. The obtained mass spectra from each sample spot are processed in Mirion to give local distributions of selected *m*/*z* signals. After AP-SMALDI MSI, the measured section (or a consecutive one) is stained using the H&E protocol

**Fig. 2:**
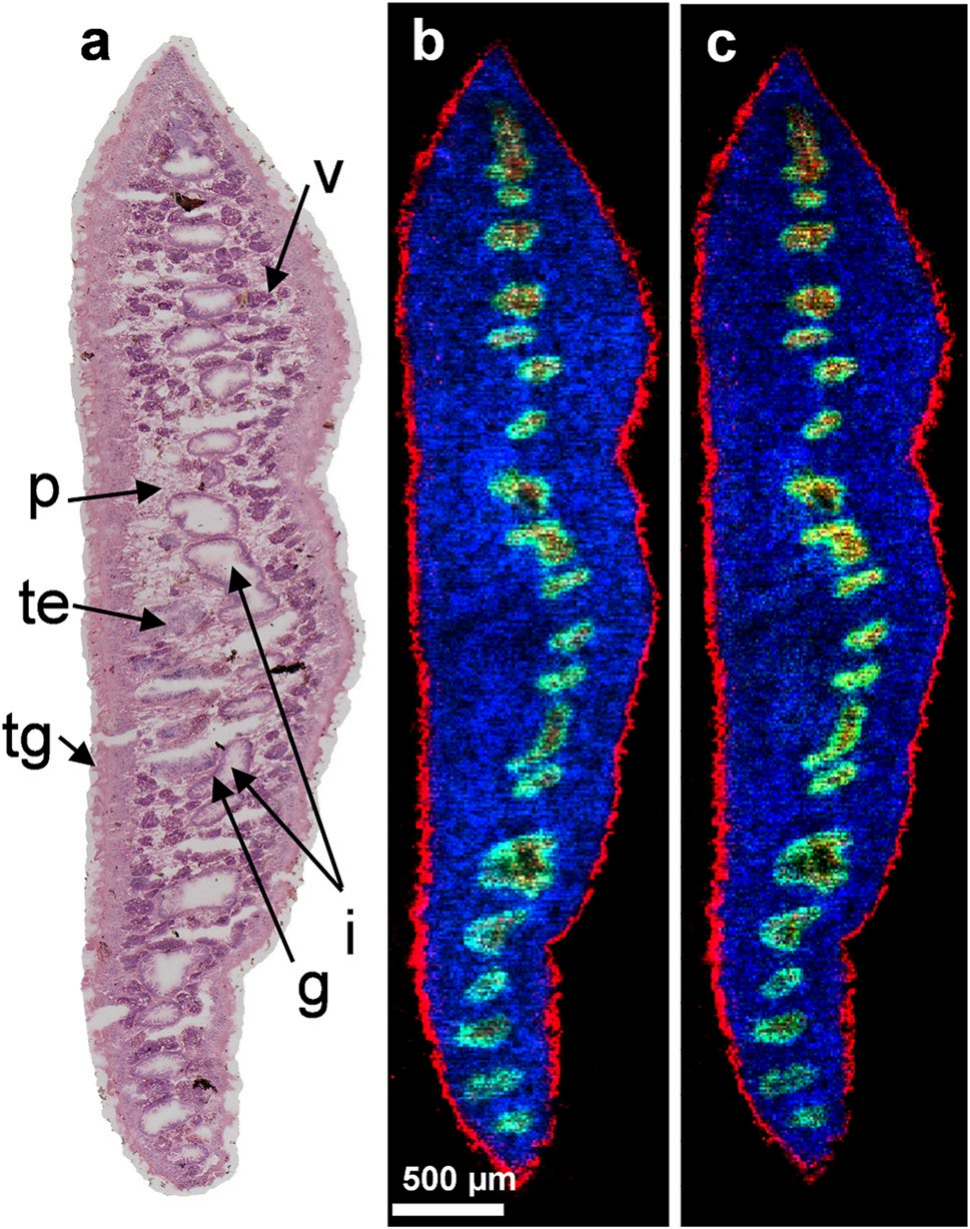
Lipid markers to discriminate tissues in *F. hepatica*. **a** H&E-stained optical image of transversal *F. hepatica* Sect. (20 µm) with organ annotations for vitellarium (v), intestine with gastrodermis (i, g), parenchyma (p), testes (te), and tegument (tg). **b** MALDI MS image of the section, showing the following signals: *m*/*z* 812.6219 (red, HexCer 38:0;O4, [M + Na]^+^), *m*/*z* 794.6035 (green, PC O-36:2, [M + Na]^+^ or PE O-39:2, [M + Na]^+^), *m*/*z* 810.5985 (blue, PC 36:1, [M + Na]^+^) used as markers for tegument, gastrodermis, and parenchyma, respectively. **c** MALDI MS image showing a second set of marker signals: *m*/*z* 828.5961 (red, PI O-33:0, [M + NH_4_]^+^ or HexCer 38:0;O4, [M + K]^+^), *m*/*z* 751.5043 (green, PA O-38:3 [M + K]^+^), *m*/*z* 796.5831 (blue, PC 35:1, [M + Na]^+^ or PE 38:1, [M + Na]^+^)

**Fig. 3:**
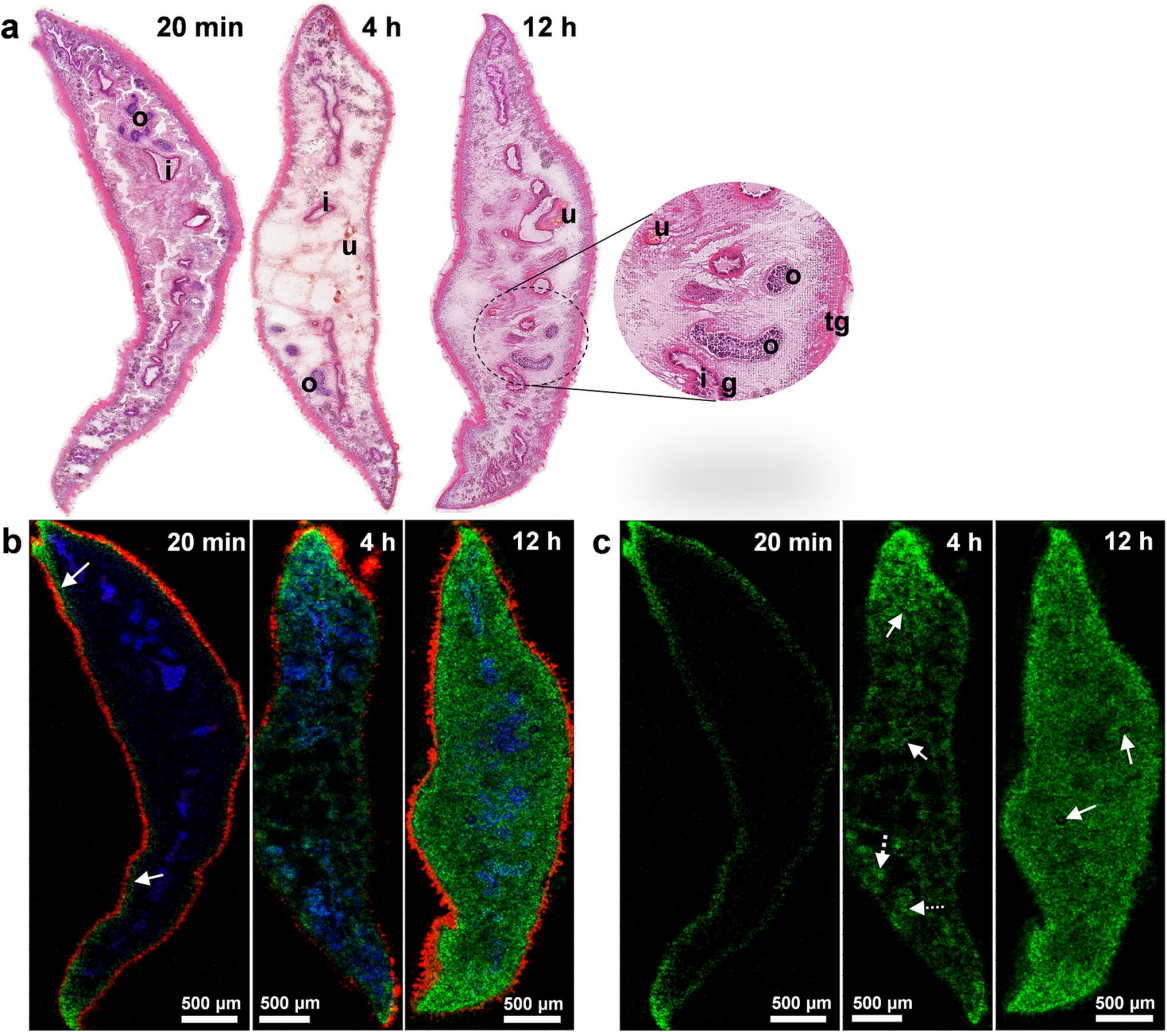
Kinetic of TCBZ uptake and distribution in *F. hepatica*. **a** H&E-stained optical images of transversal Sects. (20 µm) of liver flukes after 20 min, 4 h, and 12 h TCBZ exposure with organ annotations for intestine with gastrodermis (i, g), tegument (tg), uterus (u), and ovary (o). **b** MALDI MS RGB overlay images of the same sections showing the following signals: *m*/*z* 828.5962 (red, PI O-33:0, [M + NH_4_]^+^ or HexCer 38:0;O4, [M + K]^+^), *m*/*z* 358.9571 (green, TCBZ, [M + H]^+^), *m*/*z* 751.5039 (blue, PA O-38:3 [M + K]^+^). **c** MALDI MS single-channel images showing the color channel of the TCBZ signal (green) from **b** alone. The arrows in the MALDI MS images indicate the accumulation of TCBZ in the subtegumental area (after 20 min), the gastrodermis and ovary tissue (solid and dashed lines, respectively, after 4 h), and the TCBZ-negative eggs within the uterus (after 12 h). Images are representative for two to three independent MALDI MS measurements for each time point

**Fig. 4:**
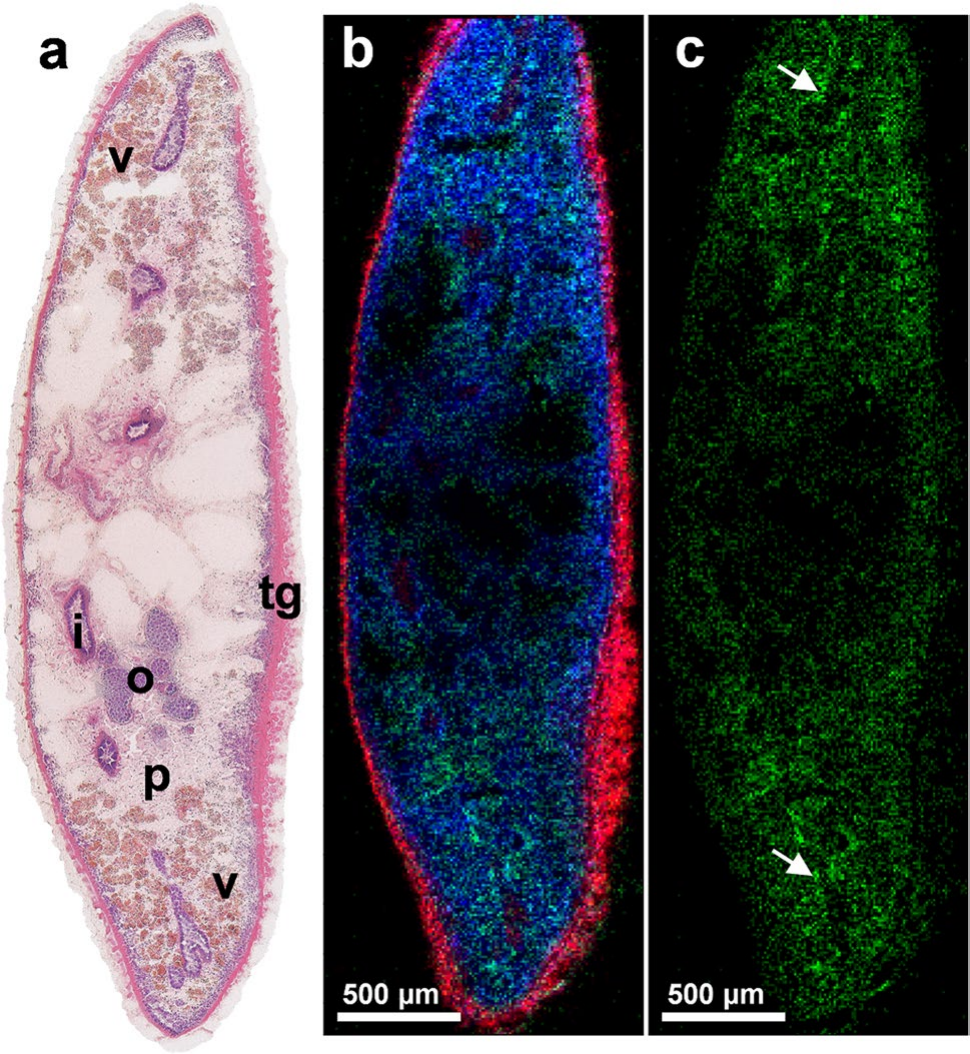
Accumulation of TCBZ sulfoxide in *F. hepatica*. **a** H&E-stained transversal *F. hepatica* section with organ annotations for vitellarium (v), intestine with surrounding gastrodermis (i), parenchyma (p), tegument (tg), and ovary (o) and corresponding **b** MALDI MS RGB image showing the following signals: *m*/*z* 828.5962 (red, PI O-33:0, [M + NH_4_]^+^ or HexCer 38:0;O4, [M + K]^+^), *m*/*z* 374.9523 (green, TCBZ-SO, [M + H]^+^), *m*/*z* 810.5996 (blue, PC 36:1), [M + Na]^+^). **c** MALDI MS single-channel image depicting the abovementioned TCBZ-SO signal only. The white arrows point to a slight accumulation of TCBZ-SO in the gastrodermis

The overall experimental workflow is presented in Fig. 1. Following drug exposure in vitro, treated worms were embedded for the preparation of appropriate tissue sections. Sectioning of worms such as *Fasciola* is generally possible in three different ways: transversal, sagittal, and frontal. The two latter options can provide a very high information content with respect to fluke organs, but these sections tend to rupture because of the large tissue area. Therefore, transversal sections, which also contain various organs and which can usually be measured within a reasonable time of 24 h with AP-SMALDI MSI (sample size 1500 ± 500 µm × 4500 ± 500 µm), were used for the experiments. Transversal sections from the middle part of the worm mainly cover the following tissues and organs: tegument (with underlying body musculature and syncytium), parenchyma, intestine with gastrodermis, vitellarium, ovary, and testes (Fig. 2a, Fig. 3a, and Fig. 4a). Next, AP-SMALDI MSI measurements were performed. To this end, a laser beam ablated sample material in grid-like patterns to obtain mass spectra from each ablated tissue spot. The mass spectra were then processed in Mirion to provide distribution patterns of selected *m*/*z* signals. Finally, the measured tissue sections were stained for tissue visualization.

### Organ assignment within the section using lipid marker signals and H&E staining

AP-SMALDI MSI is useful to investigate into which parasite tissues a drug accumulates. Yet, especially if the drug signal is weak or restricted to areas with no clearly shaped organs, it can be difficult to make organ assignments. Therefore, the identification of MS marker signals, which occur in specific tissues of the worm, is often crucial to make reliable statements on drug tropism. Markers for the tegument and the gastrodermis of the fluke are of particular interest because they represent contact areas to the host and are therefore surfaces for drug uptake (Meaney et al. 2005). Furthermore, tegumental markers clearly mark the edge of the section, which allows to discriminate tissue from embedding material as well as exteriorly localized drug from incorporated drug.

In this study, we identified two marker signals each for (1) gastrodermis and tegument (here used and referred to as tegument markers), (2) the inner tissues (mainly parenchyma, therefore referred to as parenchyma markers), and (3) the gastrodermis plus ovary tissue. Due to the *m*/*z* range of the signals, their (phospho)lipid-specific decimal places, and the low deviations between our measured masses and the theoretical masses of the LIPIDMAPS database hits, the markers can be attributed to be lipids. These lipids were either enriched or almost exclusively found in the indicated tissue types, which is evident from single color channel MS images (Suppl. Figure 1) and RGB-overlay images depicting one of the two possible marker signals for tegument, parenchyma, and gastrodermis (ovary not occurring in this particular section) within the same image (Fig. 2b and c). Further assurance of organ assignment was obtained by comparing MS images with the corresponding H&E-stained optical image (Fig. 2a). We assigned the signals to lipid bulk formulae with the help of LIPIDMAPS database (LMSD search) by using the hit(s) with the lowest deviation from our measured mass (Table 1). The tegumental marker at *m*/*z* 812.6219 was assigned to the lipid HexCer 38:0;O4 (occurring as [M + Na]^+^ ion), and the signal at *m*/*z* 828.5961 either to PI O-33:0 ([M + NH_4_]^+^) or the potassium adduct [M + K]^+^ of HexCer 38:0;O4. The marker signal for the gastrodermis and ovary tissue at *m*/*z* 794.6035 likely belonged either to PC O-36:2 or PE O-39:2 (both occurring as sodium adducts [M + Na]^+^), and the *m*/*z* 751.5043 signal was labeled to PA O-38:3 ([M + K]^+^). The parenchyma was marked by *m*/*z* 810.5985, which we identified as PC 36:1 ([M + Na]^+^), and *m*/*z* 796.5831, either corresponding to the lipid PC 35:1 or PE 38:1 (both [M + Na]^+^).

**Table 1:** Overview of lipid markers detected in *F. hepatica*. Lipid marker signals from Fig. 2 with corresponding tissue types, theoretical *m*/*z* values of the assigned lipids, the resulting *m*/*z* error between the measured and the theoretical *m*/*z* values, and bulk formulae of the annotated lipids and their ionic appearance in the MSI data

Marked tissue	Measured *m*/*z*	Theoretical *m*/*z*	Error [ppm]	Annotated lipid
Tegument/gastrodermis	812.6219	812.6222	− 0.4	HexCer 38:0;O4, [M + Na]^+^
	828.5961	828.5960 828.5962	+ 0.1 − 0.1	PI O-33:0, [M + NH_4_]^+^ or HexCer 38:0;O4, [M + K]^+^
Gastrodermis/ovary	794.6035	794.6034	+ 0.1	PC O-36:2, [M + Na]^+^ or PE O-39:2, [M + Na]^+^
	751.5043	751.5038	+ 0.7	PA O-38:3, [M + K] ^+^
Parenchyma/reproductive organs	810.5985	810.5983	+ 0.2	PC 36:1, [M + Na]^+^
	796.5831	796.5827	+ 0.5	PC 35:1, [M + Na]^+^ or PE 38:1, [M + Na]^+^

In some cases, we assigned more than one lipid candidate. This was done when more than one low-deviation hit was provided or when the lipids were isobaric. For the parenchyma marker signal at *m*/*z* 810.5985, which might correspond to the isobaric lipids PC 36:1 or PE 39:1, signal intensity was high enough to perform higher-energy collisional dissociation (HCD). In this experiment, the neutral loss of a PC lipid head group was observed, which enabled us to rule out the isobaric PE 39:1 lipid option (Suppl. Figure 2).

Due to the larger body size of *F. hepatica*, discrimination of organs by markers is significantly easier than in *S. mansoni*, a related trematode species for which we previously established AP-SMALDI MSI of the kinase inhibitor imatinib and identified lipid markers for its ovary and tegument (Kadesch et al. 2020; Mokosch et al. 2021). Therefore, AP-SMALDI-based drug-imaging experiments that aim at investigating drug tissue tropism, and lipid profiles of specific tissues are even more informative for *F. hepatica*. The six identified marker lipids allowed a very clear assignment of particular parts of the liver fluke. In a future study, we plan to determine specific lipids for other tissues as well, such as musculature, and male and female gonads in order to generate a tissue atlas of the liver fluke based on lipid markers.

### AP-SMALDI MSI reveals TCBZ tissue distribution and possible routes of uptake

To get an idea of uptake routes and tissue tropism of TCBZ in *F. hepatica*, we examined sections from flukes that were exposed to the drug in vitro for 20 min, 4 h, and 12 h, respectively. We used a test concentration of 100 µM, which affected fluke motility but was still sublethal (own observation) within the 12 h of treatment. In the MALDI MS images of tissue sections obtained for the three different exposure times, the TCBZ signal was detected at *m*/*z* 358.9571, presented as RGB overlays with two lipid marker signals, and as single-channel images in Fig. 3. Corresponding optical images of the same sections after H&E staining are given for comparison. After 20 min of TCBZ exposure, the drug was detected in the tegumental and sub-tegumental regions of the fluke, while it has spread further into the parenchyma and interior organs after 4 h. Among others, TCBZ accumulated in the gastrodermis and the ovary. After 12 h, TCBZ reached an almost uniform distribution within the entire tissue section. Eggs within the uterus, which were present in the 12 h-section, appeared to remain negative for TCBZ even after this long exposure. Root-mean-square-error (RMSE) plots of the presumed TCBZ signal depicted a single-modal peak shape, which confirmed that every green pixel in the MALDI MSI images represented TCBZ only and not additional ions of similar *m*/*z* values (Suppl. Figure 3, also depicting RMSE plots of the lipid markers and the sulfoxide).

Based on the exclusively tegumental and sub-tegumental detection of TCBZ after short incubation time, we conclude on a tegumental route of uptake. The TCBZ-positive area located underneath the tegumental lipid marker signal presumably represented the sub-tegumental muscle layers and/or the syncytium. The infiltration of TCBZ throughout the entire section area after 12 h of incubation relates well to the overall loss of worm vitality. Results from previous studies that applied mechanical ligation of the oral uptake route also suggested a tegumental uptake of TCBZ or TCBZ-SO under ex vivo conditions (Bennett und Köhler 1987; Toner et al. 2009).

The tissue distribution and uptake kinetics of TCBZ clearly differ from the kinase inhibitor imatinib that we analyzed in *F. hepatica* by AP-SMALDI MSI in a previous study (Morawietz et al. 2020). Imatinib was already detectable within the fluke section after 20 min of incubation as was TCBZ, yet imatinib was not only present in surface-near tissue, but also in parts of the intestinal region indicating an early oral drug uptake. After 4 h, imatinib preferentially accumulated in the vitellarium, while TCBZ did not show a strong selective tissue tropism. Another noticeable difference between the TCBZ and the imatinib uptake is the signal intensities. While the average intensities (of all the drug-positive pixels per tissue section) for the imatinib signal increased by more than one order of magnitude from 20 min to 12 h, the increase of the TCBZ average signal intensities within the same time period was rather negligible and intensities remained in the same magnitude. Thus, while imatinib accumulates in the parasite over time, we hypothesize that TCBZ is metabolized within the tissue in a similar speed as it is taken up, which would lead to a roughly constant TCBZ amount. Alternatively or in parallel to this, drug uptake and removal by efflux pumps might occur to a comparable extent (Mottier et al. 2006). Taken together, AP-SMALDI MSI is a useful tool to reveal differences in uptake and distribution characteristics of drugs into liver flukes.

### Detection of a TCBZ metabolite in *F. hepatica*

TCBZ-sulfoxide (TCBZ-SO) has been found as one of the major TCBZ-metabolites formed in the liver of the host animal (Moreno et al. 2014). Consequently, *F. hepatica* is intensely exposed to TCBZ-SO in an in vivo situation. Due to its high bioactivity (Stitt und Fairweather 1993), information on tissue tropism of TCBZ-SO within the parasite is of similar interest as for the parent drug. Further, it is of interest whether a conversion of TCBZ into TCBZ-SO and the further oxidized metabolite TCBZ-sulfone (TCBZ-SO_2_), which also appears to have some bioactivity (Halferty et al. 2009), occurs during in vitro exposure of the parasite. In the MSI data of TCBZ-treated flukes, we detected no significant signals of the abovementioned metabolites. Therefore, we incubated *F. hepatica* with TCBZ-SO directly for 12 h and analyzed it with AP-SMALDI MSI followed by H&E staining (Fig. 4). Similar to TCBZ, TCBZ-SO spread throughout the worm tissues after 12 h and showed a slight accumulation in the gastrodermis. These results fit to the observation that fluke motility was affected similarly as after TCBZ treatment at the same concentration (data not shown). In an untreated control section, no signals for TCBZ and TCBZ-SO were present (Suppl. Figure 4). After TCBZ-SO treatment, we found no MS signals corresponding to the second metabolite TCBZ-SO_2_.

The question whether *F*. *hepatica* is actually able to metabolize TCBZ to TCBZ-SO by itself is discussed rather controversially in the literature. Some HPLC-based studies showed the capability of the microsomal fraction of *F. hepatica* to convert TCBZ to TCBZ-SO in vitro (Mottier et al. 2004; Alvarez et al. 2005) but when whole flukes were incubated, no TCBZ-SO was detectable (Mottier et al. 2004). The conversion from TCBZ-SO to TCBZ-SO_2_, however, was demonstrated to occur within whole worms with a peak 12 h after TCBZ-SO in vitro treatment (Robinson et al. 2004). In contrast, we failed to detect TCBZ-SO_2_ possibly because the amount of parent metabolite converted into TCBZ-SO_2_ was too low to be detected. Attempts to increase sensitivity of AP-SMADI MSI measurements by using the full pixel mode of our ion source (Müller et al. 2021) and thereby increasing the pixel size to 25 µm were still unsuccessful to yield significant TCBZ-SO_2_ signals. Another possible explanation for the failure of TCBZ-SO_2_ detection is that the sulfoxide and the sulfone strongly compete with each other for the charge carriers in the ionization process with the sulfoxide having the higher affinity. The theoretical possibility that our method is unsuitable to allow detection of TCBZ-SO_2_ (e.g., because of ionization mechanisms) can be ruled out. We obtained signals of TCBZ-SO_2_ in MALDI MS measurements in standard solutions of the pure compound as [M + H]^+^ at *m*/*z* 390.9470 and [M + Na]^+^ at *m*/*z* 412.9290 in a similar intensity as the [M + H]^+^ and [M + Na]^+^ signals of the well-detectable TCBZ-SO (data not shown). It is possible that longer incubation time periods of flukes with TCBZ or TCBZ-SO would provide a detectable metabolite quantity. Future studies could involve MALDI MSI of liver flukes from TCBZ-treated animals to reveal if this method can confirm the previously suggested uptake of TCBZ metabolites produced by the host liver (Moreno et al. 2014).

## Conclusions

Knowledge on uptake mechanisms and distribution of a drug or its metabolites in tissues are key factors for understanding drug efficacy. Classical methods applied to detect drugs and their metabolites within liver flukes, such as HPLC and LC–MS, lack spatial information. Here, we demonstrate an experimental approach that overcomes existing limitations, AP-SMALDI MSI, which delivers MS data integrated into imaging data. This technique allows drawing conclusions on the uptake kinetic, route of uptake and the tissue tropism of drugs or drug candidates as we exemplified for TCBZ and one of its main metabolites in *F. hepatica*. TCBZ appeared to be taken up by the parasite’s tegumental surface within 20 min and has been found evenly distributed in most tissues after 12 h, a time period when clear reduction of vitality occurred. Furthermore, we identified lipid marker signals that allow easy orientation within the parasite sections and to accurately allocate even small accumulations of drug signals. We propose that as for TCBZ, AP-SMALDI MSI will be a useful tool to investigate different aspects of tissue distribution of other compounds, e.g., bioactive molecules. This method is not only suitable for flatworms such as *F. hepatica* and *S. mansoni* but likely also for other parasites.

## Supplementary Information

Below is the link to the electronic supplementary material.

Supplementary file1 (PDF 716 kb)

## Data Availability

All data are available as part of this article.
