# Supplementary material for: Spatial visualization of drug uptake and distribution in Fasciola hepatica using high-resolution AP-SMALDI mass spectrometry imaging
Source: Parasitol Res. 2022 Jan 24;121(4):1145–53. doi: 10.1007/s00436-021-07388-1 (PMC8986696; doi:10.1007/s00436-021-07388-1)
Supplement: Supplementary file 1 — Supplementary file1 (PDF 716 kb) [file 436_2021_7388_MOESM1_ESM.pdf]

Supplementary Information

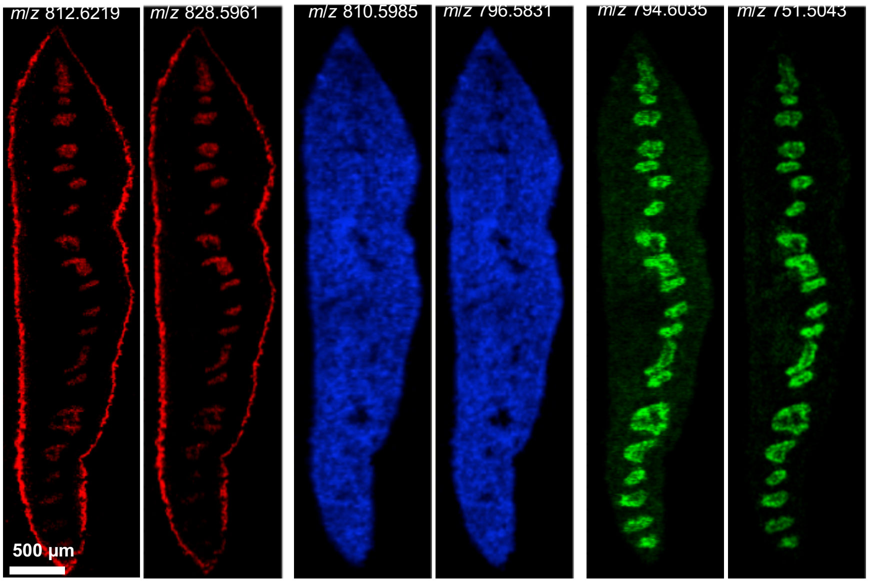

Suppl. Fig. S1 MALDI MS single-channel images of lipid markers. Single channels of the overlay image shown in Fig. 2, depicting the signals for tegument (red), parenchyma (blue), and gastrodermis (green).

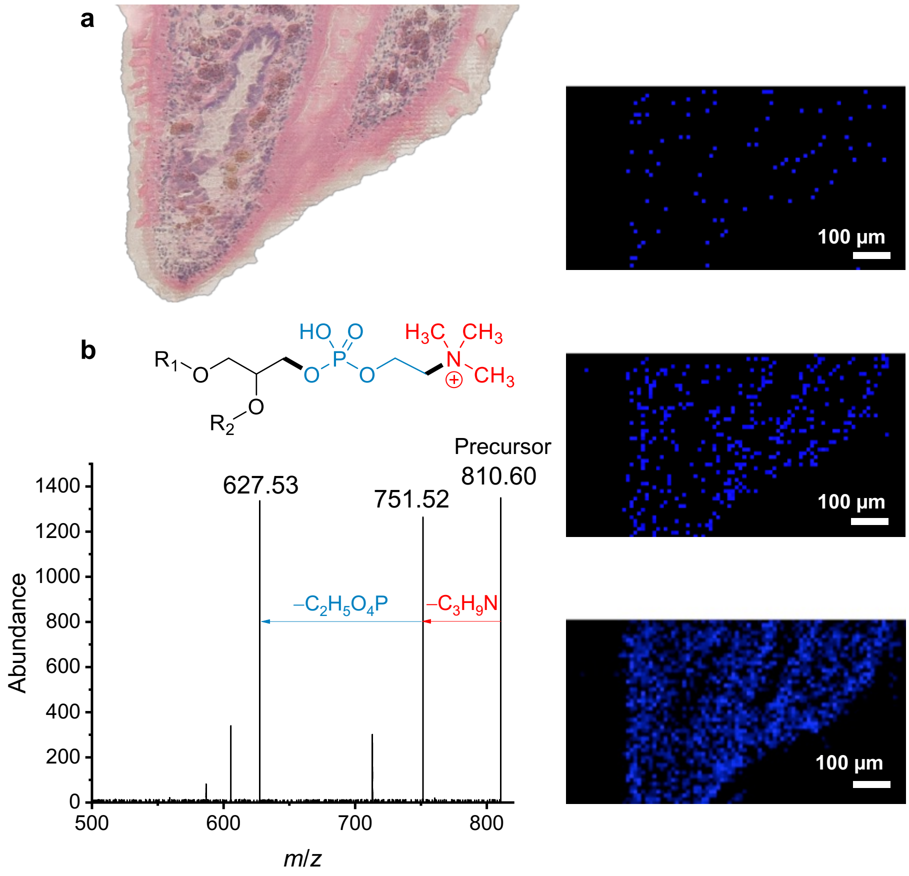

Suppl. Fig. S2 Precursor-ion fragmentation discriminates PC from PE lipid. (**a**) H&E-stained optical image of *F. hepatica* section. (**b**) Averaged HCD-MS^2 spectrum of the parenchyma tissue marker ion at *m*/*z* 810.60 (shown as generalized PC lipid structure) with annotated neutral losses of the PC lipid

head group parts (red and blue; the bold bonds are broken heterogeneously in the fragmentation process), and the two fragment ions at *m*/*z* 751.52 and *m*/*z* 627.53. (**c**) MS^2 images of the in **a** displayed section area, showing in blue the precursor ion and the two fragment ions that were detected.

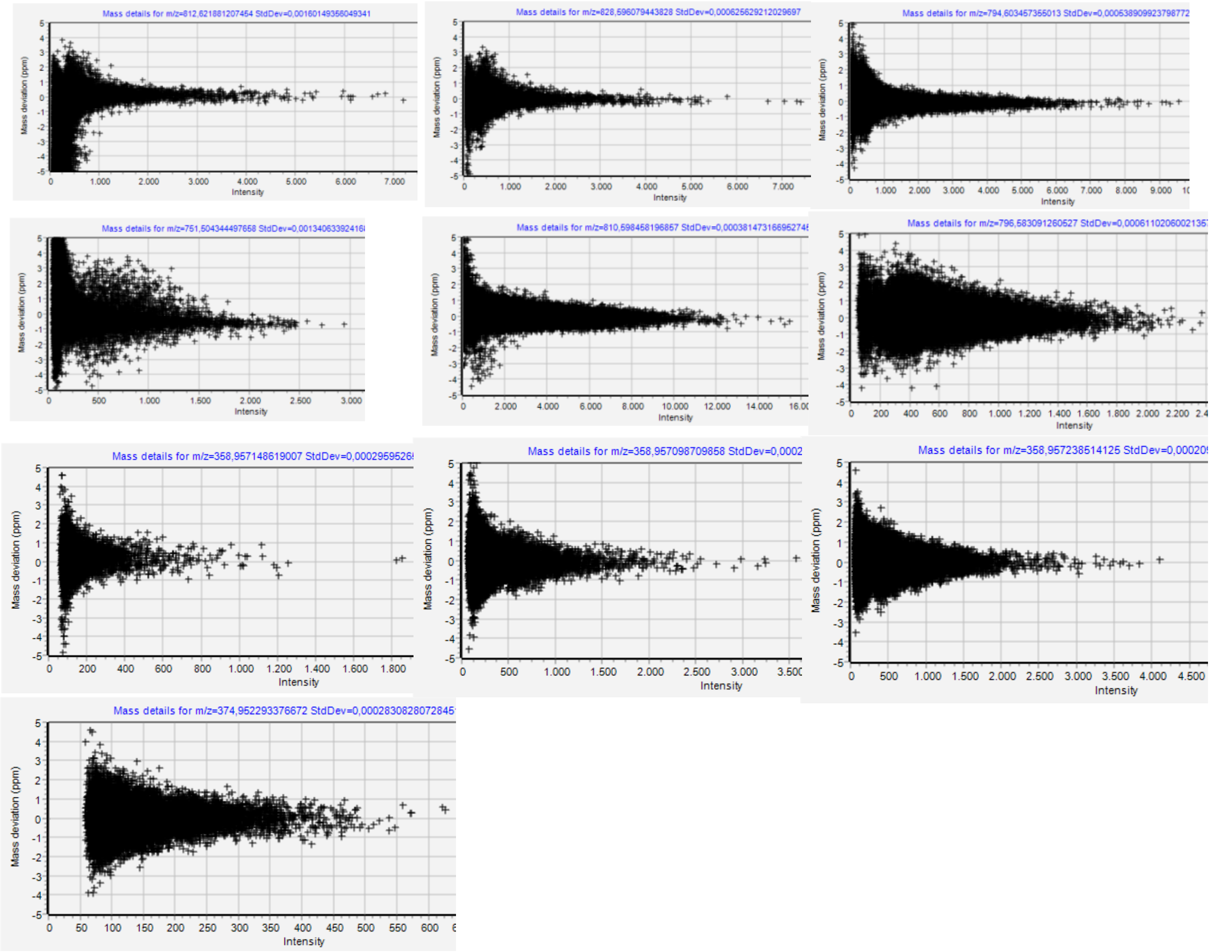

Suppl. Fig. S3 Root mean square error (RMSE) plots. The diagrams show the lipid signals and the TCBZ and TCBZ-SO signals. The single-modal peak shapes indicate that no other nearby signal contributes to the color channels in the MS images, so that every pixel represents the annotated *m*/*z* signal only. The vertical axis depicts the mass deviations in ppm, the horizontal axis the signal intensities.

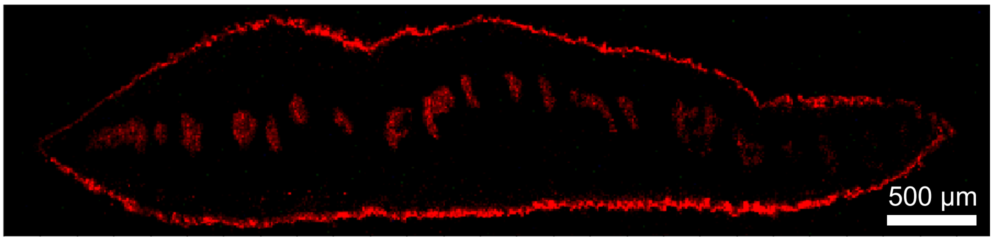

Suppl. Fig. S4 MALDI MS RGB-image of untreated control section. The red lipid signal at *m*/*z* 828.5961 (corresponding to PI O-33:0 as $[M+NH_4]^+$ or HexCer 38:0;O4 as $[M+K]^+$) indicates the outline of the untreated worm section. The green and blue channels are occupied by the TCBZ and TCBZ-SO signals, respectively (*m*/*z* 358.9566 and *m*/*z* 374.9531, both $[M+H]^+$). No significant signals are present for the drug compound and its metabolite in the control section.
